# Relative Validity of the HELIUS Food Frequency Questionnaire for Measuring Dietary Intake in Older Adult Participants of the Longitudinal Aging Study Amsterdam

**DOI:** 10.3390/nu12071998

**Published:** 2020-07-05

**Authors:** Marjolein Visser, Liset E. M. Elstgeest, Laura H. H. Winkens, Ingeborg A. Brouwer, Mary Nicolaou

**Affiliations:** 1 Department of Health Sciences, Amsterdam Public Health research institute, Faculty of Science, Vrije Universiteit Amsterdam, De Boelelaan 1085, 1081 HV Amsterdam, The Netherlands; liset.elstgeest@vu.nl (L.E.M.E.); ingeborg.brouwer@vu.nl (I.A.B.); 2 Consumption and Healthy Lifestyles Group, Wageningen University & Research, Hollandseweg 1, 6706 KN Wageningen, The Netherlands; laura.winkens@wur.nl; 3 Department of Public Health, Amsterdam UMC, Amsterdam Public Health Research Institute, University of Amsterdam, Meibergdreef 9, 1105 AZ Amsterdam, The Netherlands; m.nicolaou@amsterdamumc.nl

**Keywords:** validation, elderly, FFQ, 24-hour recall, diet assessment

## Abstract

The aim of this study was to determine the relative validity of the HEalthy LIfe in an Urban Setting (HELIUS) food frequency questionnaire (FFQ) in assessing the dietary intake of energy, nutrients, and food groups of Dutch older men and women. In 2014–2015, 88 participants of the Longitudinal Aging Study Amsterdam aged 71.9 (SD 8.6) years completed the 238-item HELIUS FFQ and three 24-hour dietary recalls. The mean group-level bias in the intakes of energy, nutrients, and food groups between the two methods was assessed, as well as Pearson’s correlation coefficients and level of agreement using quintile distribution. For the intakes of energy and macronutrients, the group-level bias was ≤5%, Pearson’s correlation coefficients were moderate to good (ranging from 0.26 for total fat to 0.72 for alcohol), and agreement was moderate to high (classification in same or adjacent quintile ranging from 63% for energy, protein, and carbohydrate to 91% for alcohol). For most micronutrients and food groups, the relative validity was moderate (Pearson’s correlation coefficients between 0.3 and 0.5), with the lowest correlations for β-carotene (0.08), vitamin B1 (0.19), fish (0.14), and grains (0.24). In conclusion, for energy and macronutrients, most micronutrients, and most food groups, the relative validity of the HELIUS FFQ to assess dietary intake in Dutch older adults was acceptable to good.

## 1. Introduction

The valid assessment of dietary intake is crucial for nutrition-related research. However, comprehensive dietary measurement methods are expensive and time-consuming and require a high commitment from participants. Self-administered food frequency questionnaires (FFQs) ask study participants about the eating frequency and portion size of a number of foods they may consume. They are suitable for estimating habitual intake over longer periods of time and are commonly used in large-scale epidemiological studies [1,2]. In comparison to other dietary intake assessment methods, FFQs are relatively inexpensive, easy, and quick to administer. Disadvantages of using FFQs are that they are subject to socially desirable answers and rely on memory, which is often reported to be a specific problem for evaluations in older adults [3]. However, no strong evidence exists that FFQs provide less valid information in community-dwelling older adults compared to younger adults [4,5].

Many Dutch FFQs have been developed and validated for use in the general, adult population excluding young (<20 years) and old (>70 years) individuals [1,6,7,8]. However, it is important to establish whether FFQs can be validly used in specific population subgroups, such as older adults. Validated tools to estimate the habitual diet of older adults are essential, as incorrect dietary information may lead to false associations of dietary factors with age-related changes in physical, emotional, and cognitive functioning.

In the Netherlands, ethnic-specific FFQs were developed for the HEalthy LIfe in an Urban Setting (HELIUS) study, with a focus on measuring the habitual diet of adults aged 18–70 years of Dutch, Surinamese, Turkish, and Moroccan populations [9]. The four questionnaires include food items reflecting those commonly consumed by the populations studied and were developed to study the association between diet and (cardiovascular) health. More information is needed on how the Dutch questionnaire performs in an older population.

The aim of this study was to assess the relative validity of the HELIUS FFQ to determine the dietary intake of energy, nutrients, and food groups in a population-based sample of older men and women.

## 2. Methods

### 2.1. Study Population and Design

The validation study is part of the Nutrition and Food-Related Behavior study, which was conducted as an ancillary study of the Longitudinal Aging Study Amsterdam (LASA) from October 2014 until April 2015. The LASA is an ongoing cohort study that aims to explore the determinants, trajectories, and consequences of physical, cognitive, emotional, and social functioning in relation to aging in the Netherlands. LASA comprises three cohorts: the first cohort (aged 55–85 years at baseline) was recruited at LASA’s start in 1992–1993, the second one (55–65 years) in 2002–2003, and the third one (55–65 years) in 2012–2013. Details on the sampling and data collection procedures have been published and described elsewhere [10,11]. The LASA study was conducted according to the guidelines laid down in the Declaration of Helsinki and was approved by the ethical committee of the VU University Medical Centre (Amsterdam, the Netherlands). All participants provided written informed consent.

Dietary information was collected by the Dutch version of the HELIUS FFQ for 1439 participants. Of the participants who completed the FFQ and had indicated to be willing to participate in a supplementary study on nutrition, 97 were randomly selected for 24-hour dietary recalls, stratified for type of questionnaire (paper or online) and receipt date of the FFQ. Three 24-hour dietary recalls were collected for 92 participants (response rate 94.8%). FFQ data of 3 of these 92 participants and 24-hour recall data of 1 participant were excluded because of implausibly high energy intakes (>4000 kcal for men and >3500 kcal for women) [12]. The analytic sample of this validation study therefore comprised 88 participants.

### 2.2. Food Frequency Questionnaire

The Dutch version of the HELIUS study [13] FFQ was used to measure food intake. HELIUS researchers at the Amsterdam UMC, location AMC (Academic Medical Center)developed the FFQ in collaboration with the National Institute for Public Health and the Environment (RIVM) and the Wageningen University. The selection of the food items included was based on (1) the percentage distribution of that food item with respect to the absolute nutrient intake and its percentage contribution to the variance in absolute nutrient intake and (2) comparability with the FFQs for different ethnic groups. Nutritional values for the food lines were based on the intake data from a random sample of 2997 Dutch citizens aged 18–69 years (among which, 832 were aged 51 to 69 years) from the Dutch National Food Consumption Survey 2007–2010 [14]. Details regarding the development of the FFQ are described elsewhere [9]. Using the paper or online questionnaire, the participants reported the eating frequency and portion size of 238 food items they might have consumed over the past four weeks. The estimation of portion sizes was facilitated by using relevant units (e.g., slice of bread) or household measures (e.g., tablespoons) and by using 11 sets of 5 or 6 pictures of a standard plate indicating different portion sizes (for butter, cheese, and meat on bread, pasta, rice, legumes, French fries, cooked potatoes, cooked vegetables, salad, and meat). Average daily energy and (macro- and micro-) nutrient intakes were calculated by multiplying the frequency of consumption by the consumed amounts and nutrient content per item using the Dutch Food Composition Table 2011 [15]. A total of 17 food groups were formed by adding the consumed amounts of individual foods based on similarity in nutrient profile and culinary use. In total, 1439 participants completed the FFQ. Participants with more than 10 missing items (N = 18) and those with implausibly high energy intakes (N = 22) were excluded, leaving a total of 1399 participants with complete FFQ data.

### 2.3. 24-Hour Dietary Recalls

Three 24-hour dietary recalls were collected by telephone (on two weekdays and one weekend day). Specifically, trained researchers telephoned the participants unexpectedly to recall their food intake of the previous day covering all foods and beverages consumed from waking up until the next morning. To ensure consistency and quality of data, a standardized interview protocol, a manual, and a registration form were used. The weight of the reported food products was estimated by the respondent (used portion of the total packaging) or by use of a portion size booklet, the measured content of commonly used kitchenware, or used recipes. Special diets (e.g., energy-restricted) and eating moments (ranging from before breakfast (1) to after dinner (7)) were also recorded. Before the interviews, the participants were sent a portion size booklet with color pictures of various food products (ranging from a buttered slice of bread to a plate with vegetables or pasta sauce) using different portion sizes. They were also instructed to measure the content of frequently used kitchenware, such as glasses, cups, bowls, and serving spoons. After interviewing and registering the daily food intake, food items were encoded and classified using the Dutch Food Composition Table 2011 [15] from which consumed amounts of nutrients and food products were obtained. One participant for whom the mean of three 24-hour dietary recalls indicated an implausibly high energy intake was excluded.

### 2.4. Descriptive Variables

Age, sex, marital status, education level, smoking status, number of chronic diseases, waist circumference, and cognitive status were assessed. Data on age and sex were derived from the municipal registries. Data on all other descriptive variables were obtained from the regular LASA 2011–2013 examination wave. Marital status was categorized into three groups: married/registered partnership, never married, widowed/divorced. The highest completed level of education was divided into low (elementary school or less), middle (general secondary, intermediate vocational, intermediate general, and lower vocational education), and high (university education, college, or higher vocational). Smoking status was self-reported and categorized into never, former, and current smoker. Self-reported number of chronic diseases included major somatic diseases: asthma/chronic obstructive pulmonary disease, cardiac disease, peripheral arterial disease, diabetes mellitus, cerebrovascular accident/stroke, osteoarthritis/rheumatoid arthritis, cancer, hypertension, and other chronic diseases. Waist circumference (cm) was measured twice to the nearest 0.1 cm in the standing position, midway between the lower rib and the iliac crest, after a normal expiration, and the mean was calculated. Elevated waist circumference was defined as a waist circumference ≥ 94 cm for men and ≥80 cm for women. General cognitive functioning was measured with the Mini-Mental State Examination (MMSE), whose score ranges from 0 to 30 [16].

### 2.5. Statistics

The characteristics of the 88 validation study participants (those with an FFQ as well as three 24-hour dietary recalls and no implausibly high energy intake) and all 1399 participants of the LASA study (those with complete FFQ data and no implausibly high energy intake) were compared using Student’s t or Chi-square tests. All further data analyses were performed in the validation study participants only. The average intake of energy, nutrients, and food groups from the three 24-hour dietary recalls was used as a reference for dietary intake. Mean bias (%) in the energy, nutrient, and food group intake estimates of the FFQ versus the reference method was calculated as (mean intake FFQ/mean intake 24-hour dietary recalls) × 100%. The relative validity of the FFQ for assessing the intake of energy, nutrients, and food groups was investigated using the Pearson’s correlation coefficient (with 95% confidence interval). We used log-transformed intake data for energy, macro-, and micronutrients. The normality of the distributions of food group data was not improved by transformation (log or square-root transformations); however, Pearson and Spearman’s correlations were similar. The attenuation factor was estimated as the slope in the linear regression of the intake estimates from the 24-hour dietary recalls on the intake estimates from the FFQ. The de-attenuated correlation coefficient (with 95% confidence interval) was estimated as the correlation coefficient between the FFQ and the 24-hour dietary recalls divided by the square root of the intra-class correlation coefficient (ICC) of the three 24-hour dietary recalls [7]. The agreement between intake data from the FFQ and those from the average of the three 24-hour dietary recalls was evaluated by classifying the participants according to their distributions into quintiles of energy, nutrients, and food groups for each method. The percentages of agreement (classification in the same or adjacent quintile) and disagreement (classification in extreme quintiles) were estimated. The level of agreement in energy and macronutrient intake estimates between the two methods was also evaluated by the Bland–Altman method by building scatterplots with the mean intake of the two methods on the *x*-axis and the absolute difference in intake (FFQ minus 24-hour dietary recalls) on the *y*-axis and by visually inspecting these plots. Data were analyzed using SPSS version 24 (SPSS Inc. Chicago, IL, USA); *p*-values < 0.05 were considered statistically significant.

## 3. Results

The characteristics of the validation sample as well as those of all LASA participants with FFQ data are shown in Table 1. The mean age of the validation sample was 71.9 (SD 8.6) years and ranged from 58 to 88 years, 52.7% were female, and all participants had an MMSE score of 24 or higher. The characteristics of the validation sample (N = 88) did not differ from those of the total sample with FFQ data (N = 1399). There were also no relevant differences between the two samples regarding energy, macronutrient, mono/di-saccharides, and dietary fiber intake as assessed by the FFQ (Table S1).

Table 2 shows the mean energy, nutrient, and food group intake from the FFQ and the three 24-hour dietary recalls and the group-level bias between the two methods. Group-level bias was small for the intake of energy, protein, fat, carbohydrates, and alcohol, and for the intake of dietary fiber, (haem and non-haem) iron, and water. Relative to the 24-hour dietary recalls, the FFQ overestimated the intake of animal protein, polyunsaturated fatty acids (PUFA), monounsaturated fatty acids (MUFA), n-3 fatty acids, n-6 fatty acids, alpha linolenic acid (ALA), eicosapentaenoic acid (EPA), docosahexaenoic acid (DHA), mono/di-saccharides, and all other minerals and all vitamins compared to the 24-hour dietary recalls. The FFQ did not underestimate the intake of any of the nutrients. For the food groups, group-level bias was small for vegetables, grains, bread, and sugar/sweets. The FFQ overestimated the intakes of fish, eggs, dairy products, fruit, nuts/seeds, and legumes, whereas it underestimated the intakes of meat, potatoes, alcoholic beverages, non-alcoholic beverages, cakes/cookies, fast food/snacks, and soups compared to the 24-hour dietary recalls.

Table 3 shows the Pearson’s correlation coefficient, the attenuation factor, and the de-attenuated correlation coefficient between the FFQ and the 24-hour dietary recall for the intake of energy, nutrients, and food groups. For energy, macronutrients, and micronutrients, most Pearson’s correlation coefficients were between 0.3 and 0.5. For the macronutrients, the Pearson’s correlation coefficients were the lowest for MUFA (0.20), EPA (0.21), and DHA (0.21) and the highest for mono/di-saccharides (0.53) and alcohol (0.72). For the micronutrients, the Pearson’s correlation coefficients were the lowest for β-carotene (0.08) and vitamin B1 (0.19) and the highest for haem iron (0.50) and vitamin B12 (0.50). For the food groups, most Pearson’s correlation coefficients were generally between 0.3 and 0.4. They were the lowest for fish (0.14) and grains (0.24) and the highest for non-alcoholic beverages (0.70) and alcoholic beverages (0.78). The attenuation factors varied from 0.03 for β-carotene and 0.06 for DHA to 0.56 for vitamin C and 0.65 for alcohol.

The agreement between the FFQ and the three 24-hour dietary recalls, based on the quintile distributions of the intakes of energy, nutrients, and food groups, is also shown in Table 3. Agreement was high (i.e., <3% in extreme quintiles) for energy, vegetable protein, ALA, total carbohydrates, polysaccharides, mono/di-saccharides, and alcohol, as well as for calcium, iron, non-haem iron, vitamin B2, vitamin B6, vitamin B12, magnesium, and water. Regarding the food groups, agreement was high for vegetables, bread, alcoholic beverages, non-alcoholic beverages, and sugar/sweets. Agreement was poor (i.e., >10% in extreme quintiles) for fish and legumes only.

**Figure 1:**
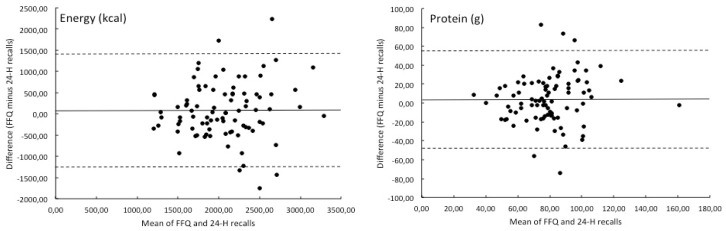

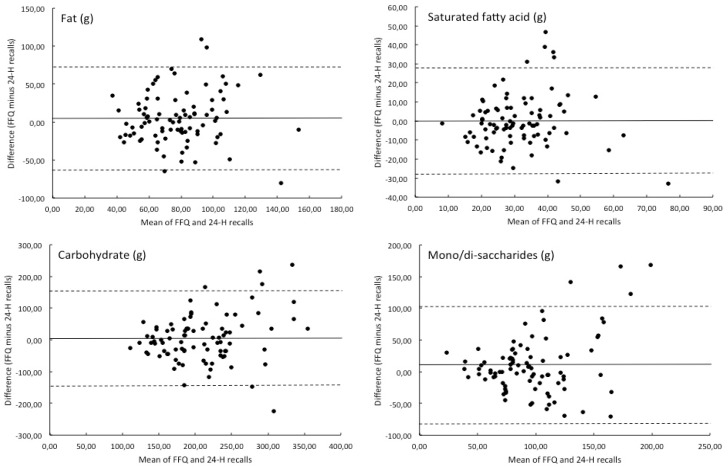
Bland–Altman plots of intake of energy, total protein, total fat, saturated fatty acids, total carbohydrates, and mono/di-saccharides assessed by the HEalthy LIfe in an Urban Setting (HELIUS) FFQ versus the mean of three 24-hour dietary recalls (24-H recalls) for 88 validation study participants of the Longitudinal Aging Study Amsterdam.

Using the Bland–Altman method, the level of discrepancy was estimated with 95% confidence intervals of agreement for the intake of energy and macronutrients only (Figure 1). The mean difference in intake between the FFQ and the three 24-hour dietary recalls (with 95% confidence interval) was 76 (−1255;1408) for energy (kcal), 3.6 (−47.6;54.8) for total protein (g), 4.9 (−62.7;72.5) for total fat (g), −0.1 (−27.9;27.7) for saturated fatty acids (g), 4.7 (−145.9;155.3) for total carbohydrates (g), and 10.3 (−82.5;103.1) for mono/di-saccharides (g). Based on visual inspection of the Bland–Altman plots, there was no indication for an increase or decrease in the intake difference between the two methods with higher mean intake values.

## 4. Discussion

In this study, we investigated the relative validity of the HELIUS FFQ to estimate the intake of energy, nutrients, and food groups in participants of the LASA study aged 58 to 88 years. For energy and macronutrients, the group-level bias was small, the Pearson’s correlation coefficient was moderate to good, and the agreement based on quintile intakes was moderate to high, implying that the FFQ is able to rank older adults according to their dietary intake of energy and macronutrients. For most micronutrients and most food groups, but not for all, the relative validity was moderate (Pearson’s correlation coefficient between 0.3 and 0.5). For all micronutrients and most food groups (except for fish and legumes), the agreement based on quintile intakes was moderate to high, indication that the FFQ can also be used to rank older adults according to their micronutrient and food group intake. These results suggest that the HELIUS FFQ can be used to estimate the intake of energy and macronutrients and of most micronutrients and food groups in older adults.

Compared to three 24-hour dietary recalls, the FFQ overestimated the intake of fish, eggs, dairy products, fruit, nuts/seeds, and legumes. The poor estimation of fish intake was also reflected in the relatively low Pearson’s correlation coefficients for fish (0.14), EPA, and DHA (0.21 and 0.21) and the poor quintile agreement for EPA (9% in extreme quintile). It is striking that overestimation was observed in particular for food groups (i.e., fish, eggs, nuts/seeds, legumes) for which the average daily intake is generally low in older Dutch adults [17] and consumption is infrequent [18]. Using the mean of three 24-hour dietary recalls may not have been appropriate to capture the usual intake of these food groups and nutrients such as EPA, DHA, and beta-carotene, and therefore, no clear conclusions can yet be drawn regarding the validity of the FFQ to estimate the intake of these food groups and nutrients. Future studies using for example nutrient blood concentrations or a higher number of 24-hour dietary recalls per person are needed to validate the estimated intake of the above-mentioned food groups and nutrients by the FFQ in older adults. Until then, the estimated intakes of these specific food groups and nutrients by the FFQ should be carefully interpreted.

It has been suggested that the FFQ method is less suitable for older adults, as it heavily relies on memory. We therefore compared the relative validity of the HELIUS FFQ in older adults as observed in this study to the relative validity of other Dutch FFQs observed in younger adults. The correlation coefficients for the intake of macronutrients for the FFQ and three 24-hour dietary recalls (protein 0.39, fat 0.26, carbohydrates 0.41, and alcohol 0.72) observed in our study were comparable to or only slightly lower than those from two recent Dutch FFQs validation studies conducted in younger adults (protein 0.38–0.51, fat 0.30–0.39, carbohydrates 0.41–0.70, and alcohol 0.77–0.78) [7,8]. These comparisons indicate that the relative validity of the HELIUS FFQ in older adults is comparable to the relative validity of other Dutch FFQs in younger adults, which suggests that the FFQ in general is as valid for application in older adults as it is for younger adults to estimate dietary intake.

The observed Pearson’s correlation coefficients for nutrients are very similar to those of FFQ validation studies conducted in older adults that also used multiple 24-hour dietary recalls as the reference method [19,20]. For example, their coefficients for energy, protein, fat, calcium, and fiber, respectively, ranged from 0.22 to 0.39, 0.30 to 0.41, 0.25 to 0.58, 0.38 to 0.54, and 0.29 to 0.50. FFQ validation studies in older adults using multiple dietary records as the reference method also showed similar results (0.19–0.40, 0.19–0.45, −0.01–0.47, 0.35–0.49, 0.49–0.55) [21,22,23,24]. For food groups, a Spanish study conducted in adults aged 55–80 years and using three-day dietary records as the reference method, reported higher correlation coefficients for meat (0.61), fish (0.42), fruit (0.57), and vegetables (0.70) compared to the current study, which could potentially be explained by their much higher intakes of these food groups [24]. Overall, these comparisons show that the validity of the FFQ is fairly similar to that of FFQs used for older adults from other countries.

The strength of our study is that we specifically included a sample of older men and women. Previous studies investigating the relative validity of Dutch FFQs excluded participants older than 70 years of age [1,6,7] or had a much lower mean age of the study sample [8]. Our study fills an important knowledge gap, showing that the HELIUS FFQ can also be used to estimate dietary intake in older persons. A second strength is that our validation sample was representative of the total LASA sample with complete FFQs with regard to general demographic, lifestyle, and health characteristics, as well as with regard to the intakes of energy, macronutrients, and dietary fiber. This observation suggests that the results of this validation study can be extrapolated to all LASA participants. Thirdly, participants in the validation study completed a total of three 24-hour dietary recalls in order to take day-to-day variation into account and because using the mean of three recalls allows obtaining results more highly correlated with the true usual intakes of energy and protein compared to using just one or two 24-hour dietary recall(s) [25]. Finally, the average consumption of energy, nutrients, and food groups from the three 24-hour dietary recalls was used as a reference for dietary intake. In contrast to the FFQ, this method used a telephone interview and was thereby less affected by visual or physical limitations of older adults compared to the completion of the FFQ (on paper or online), and it does not depend on long-term memory and the capability of older participants to calculate their average intake over a time period of four weeks. This will lower the correlation between measurements errors of both methods and decrease the likelihood of inflating the relative validity.

Some limitations of our study should also be addressed. The FFQ was obtained first (with a reference period of the past 4 weeks), followed by three 24-hour recalls obtained within 6 weeks after completion of the FFQ. Even though the intake data of both methods were obtained within a relatively small time frame, the time periods during which the food intake was assessed by both methods did not overlap, likely reducing the reported relative validity. In addition, no objective methods such as urinary or serum nutrient concentrations or total energy expenditure using the doubly labelled water method were available to validate the FFQ. However, the repeated 24-hour dietary recall method is considered the next best alternative reference method when biomarkers are not available [26]. The FFQ was originally developed for adults aged 18–69 years. Portion sizes might be smaller in older adults compared to younger adults, and the nutritional values assigned to the food lines may be different between older and younger adults [27], which could potentially impact the validity of the FFQ in older adults. Because the FFQ used relevant units, household measures, as well as pictures to indicate portion size and because portion size estimation is similar in younger and older adults [28] and using pictures is a valid method to estimate portion size by older persons [29], this impact is expected to be limited. Furthermore, previous studies conducted in the Netherlands showed that the selected foods for the food lists of an FFQ for older adults were similar to those identified for younger adults [5]. Although using a telephone interview for the 24-hour dietary recalls has some advantages as discussed above, a face-to-face interview might have been better for older adults suffering from hearing problems. The telephone interview also did not allow the use of food models; however, participants were instructed to measure the content of frequently used kitchenware before their first recall and were sent a picture book to facilitate the estimation of portion sizes. A final limitation is that the number of participants in the current study was lower in comparison to those of other FFQ validation studies (median 110 subjects) [30] and the recommended number [12].

In conclusion, the results of this validation study show that the relative validity of the HELIUS food frequency questionnaire to assess dietary intake in older adults was acceptable to good for energy and macronutrients and for most micronutrients and most food groups. The relative validity is comparable to the reported relative validity of other Dutch FFQs versus 24-hour dietary recalls in younger adults.

## Figures and Tables

**Table 1:** Characteristics of the 88 validation study participants (those with a completed food frequency questionnaire as well as three 24-hour dietary recalls) and all 1399 participants (those with complete food frequency questionnaire data) of the Longitudinal Aging Study Amsterdam.

Characteristic	Validation Sample	Total FFQ Sample
N	88	1399
Age (year) (mean (SD))	71.9 (8.6)	69.5 (8.6)
Age (year) (minimum-maximum)	58.0–88.4	56.8–101.8
Female (%)	54.5	52.7
Marital status (%)		
Married or registered partnership	64.8	71.8
Not married	8.0	7.6
Widowed or divorced	27.2	20.6
Educational level (%)		
Low education	12.5	12.2
Middle education	70.5	58.4
High education	17.0	29.4
Smoking status (%)		
Never smoker	30.7	26.9
Former smoker	56.8	58.2
Current smoker	10.2	11.3
Missing	2.3	3.6
Number of chronic diseases (%)		
No chronic diseases	11.4	15.9
One chronic disease	25.0	28.0
≥2 chronic diseases	63.6	56.1
Elevated waist circumference (%) *	81.8	80.5
MMSE score (mean (SD))	28.4 (1.5)	28.3 (1.9)

FFQ = food frequency questionnaire, SD = standard deviation, MMSE = Mini-Mental State Examination. All group differences *p* > 0.05. * Defined as a waist circumference ≥94 cm for men and ≥80 cm for women.

**Table 2:** Mean energy, nutrient, and food group intake from the food frequency questionnaire and three 24-hour dietary recalls and group level bias between the two methods in the 88 validation study participants of the Longitudinal Aging Study Amsterdam.

	Absolute Intake				Group-Level Bias
	FFQ		24-Hour Dietary Recalls		
	Mean	SD	Mean	SD	%
Energy (kcal)	2097	579	2021	533	3.8
Macronutrients					
Protein (en%)	15.6	2.9	15.5	2.7	0.8
Total (g)	81.1	24.2	77.5	21.7	4.7
Vegetable (g)	30.4	9.8	30.2	10.9	0.4
Animal (g)	50.7	19.9	47.3	15.1	7.3
Fat (en%)	34.2	6.1	33.3	6.2	2.6
Total (g)	80.5	28.9	75.6	27.1	6.5
SFA (g)	31.3	13.2	31.4	12.8	−0.3
PUFA (g)	29.8	11.3	14.1	6.7	7.0 *
MUFA (g)	17.2	7.8	27.8	10.9	21.4
n-3 FA (g)	2.3	1.1	1.9	0.9	20.5 *
n-6 FA (g)	14.5	6.7	11.9	5.89	22.0 *
ALA (g)	2.0	0.9	1.7	0.82	20.9 *
EPA (g)	0.1	0.1	0.1	0.1	31.3 *
DHA (g)	0.1	0.1	0.1	0.2	16.8 *
Carbohydrates (en%)	40.6	7.8	41.5	7.0	−2.2
Total (g)	212.0	72.7	207.3	58.8	2.3
Polysaccharides (g)	109.6	38.9	115.2	39.9	−4.8
Mono/di-saccharides (g)	102.2	47.3	91.9	35.4	11.2
Dietary fibre (g)	22.2	7.0	21.5	7.1	3.5
Alcohol (en%)	4.8	6.2	5.0	5.6	−3.7
Alcohol (g)	14.1	18.3	15.1	18.7	−6.6
Micronutrients					
Calcium (mg)	1060.8	418.0	957.6	336.0	10.8
Iron (mg)	11.1	3.0	10.9	3.5	1.9
Haem (mg)	1.2	0.7	1.1	0.8	4.0
Non-haem (mg)	9.9	2.7	9.8	3.4	1.6
Retinol (µg)	851.5	882.0	708.9	553.1	20.1
β-carotene (µg)	2393.3	1472.0	2250.2	2431.7	6.4
Vit B1 (mg)	1.1	0.4	1.0	0.5	5.4
Vit B2 (mg)	1.5	0.6	1.4	0.5	8.1
Vit B6 (mg)	1.7	0.6	1.6	0.6	9.4
Vit B12 (µg)	5.8	4.2	4.7	3.2	23.8 *
Vit C (mg)	139.4	70.7	101.8	58.5	36.9 **
Vit D (µg)	3.8	2.1	3.3	2.1	15.5 *
Folate (µg)	282.9	80.6	252.4	80.7	12.1 *
Phosphorous (mg)	1526.7	447.7	1407.1	409.0	8.5 *
Magnesium (mg)	360.6	93.0	340.0	105.0	6.1 *
Zinc (mg)	11.0	3.5	10.4	3.1	5.5
Water (g)	2439.9	707.3	2502	849	−2.5
Food groups (g)					
Meat	86.9	51.7	100.1	59.5	−13.2 *
Fish	20.3	18.8	16.5	29.7	23.0
Eggs	16.2	17.0	13.2	17.2	22.4
Dairy products	363.5	269.4	295.0	168.9	23.2 *
Fruit	227.2	166.0	144.0	111.8	57.8 **
Vegetables	155.6	93.3	160.7	117.1	−3.2
Nuts/seeds	13.2	15.7	9.3	16.6	41.5 *
Potatoes	86.5	71.8	97.7	103.5	−11.4
Legumes	15.5	25.6	9.8	45.3	59.2 **
Grains	45.6	36.2	46.9	54.4	−2.8
Bread	124.6	59.4	120.9	47.7	3.0
Alcoholic beverages	151.9	197.9	173.2	245.5	−12.3
Non-alcoholic beverages	1335.1	580.1	1526.3	710.9	−12.5 *
Sugar/sweets	30.6	27.8	29.3	25.4	4.6
Cakes/cookies	27.0	22.1	40.6	30.5	−33.4 **
Fast food/snacks	20.6	23.6	37.4	55.7	−45.0
Soups	51.7	65.6	55.9	82.2	−7.6

en% = expressed as a percentage of total energy intake; SFA = saturated fatty acids; PUFA = polyunsaturated fatty acids; MUFA = monounsaturated fatty acids; ALA = alpha linolenic acid; EPA = eicosapentaenoic acid; DHA = docosahexaenoic acid; Vit = vitamin. * *p* < 0.05; ** *p* < 0.001.

**Table 3:** Pearson’s correlation coefficient, attenuation factor, and de-attenuated correlation coefficient of energy, nutrients, and food groups intake between the food frequency questionnaire and three 24-hour dietary recalls and agreement between the two methods based on quintiles of energy, nutrient, and food group intake in the 88 validation study participants of the Longitudinal Aging Study Amsterdam.

	Pearson’s Correlation Coefficient *		Attenuation Factor		De-Attenuated Correlation Coefficient	Agreement	
						Same or Adjacent Quintile	Extreme Quintile
		95% CI		95% CI		%	%
Energy (kcal)	0.32	0.13; 0.53	0.31	0.08; 0.53	0.46	63	2
Macronutrients							
Protein (en%)							
Total (g)	0.39	0.12; 0.62	0.43	0.21; 0.65	0.54	63	5
Vegetable (g)	0.49	0.31; 0.64	0.47	0.30; 0.63	0.65	77	2
Animal (g)	0.41	0.18; 0.59	0.46	0.23; 0.73	0.82	73	3
Fat (en%)							
Total (g)	0.26	0.06; 0.47	0.28	0.07; 0.50	0.41	72	7
SFA (g)	0.43	0.17; 0.66	0.45	0.25; 0.64	0.67	74	5
PUFA (g)	0.26	0.07; 0.46	0.28	0.06; 0.51	0.38	72	7
MUFA (g)	0.20	0.04; 0.34	0.19	0.02; 0.40	0.34	57	7
n-3 FA (g)	0.32	0.11; 0.52	0.32	0.09; 0.56	0.51	66	5
n-6 FA (g)	0.28	0.06; 0.47	0.29	0.08; 0.51	0.41	65	6
ALA (g)	0.32	0.09; 0.49	0.29	0.06; 0.51	0.47	60	2
EPA (g)	0.21	0.01; 0.40	0.14	0.01; 0.27	0.46	68	9
DHA (g)	0.21	0.03; 0.45	0.06	0.08; 0.19	0.61	60	6
Carbohydrates (en%)							
Total (g)	0.41	0.24; 0.58	0.45	0.20; 0.69	0.62	63	1
Polysaccharides (g)	0.51	0.35; 0.66	0.46	0.28; 0.65	0.73	75	0
Mono/di-saccharides (g)	0.53	0.35; 0.65	0.54	0.28; 0.81	0.71	69	2
Dietary fibre (g)	0.43	0.24; 0.59	0.43	0.24; 0.62	0.57	69	3
Alcohol (en%)							
Alcohol (g)	0.72	0.57; 0.87	0.65	0.50; 0.81	0.93	91	1
Micronutrients							
Calcium (mg)	0.45	0.23; 0.62	0.47	0.22; 0.71	0.61	73	1
Iron (mg)	0.43	0.23; 0.60	0.41	0.23; 0.57	0.60	67	2
Haem (mg)	0.50	0.20; 0.69	0.34	0.14; 0.54	1.12	65	3
Non-haem (mg)	0.47	0.28; 0.63	0.40	0.25; 0.55	0.61	74	1
Retinol (µg)	0.42	0.17; 0.56	0.34	0.01; 0.68	1.08	69	5
β-carotene (µg)	0.08	0.13; 0.28	0.03	0.10; 0.16	0.15	55	6
Vit B1 (mg)	0.19	0.03; 0.38	0.12	0.05; 0.29	0.46	68	6
Vit B2 (mg)	0.45	0.25; 0.60	0.51	0.27; 0.75	0.60	56	2
Vit B6 (mg)	0.27	0.05; 0.47	0.27	0.05; 0.48	0.58	65	2
Vit B12 (µg)	0.50	0.28; 0.67	0.44	0.18; 0.71	0.96	67	2
Vit C (mg)	0.44	0.29; 0.61	0.56	0.33; 0.79	0.96	72	6
Vit D (µg)	0.44	0.11; 0.80	0.22	0.01; 0.42	0.72	64	3
Folate (µg)	0.34	0.14; 0.51	0.34	0.13; 0.54	0.50	64	5
Phosphorous (mg)	0.34	0.14; 0.61	0.40	0.18; 0.62	0.43	69	6
Magnesium (mg)	0.46	0.26; 0.62	0.41	0.24; 0.58	0.58	67	1
Zinc (mg)	0.36	0.13; 0.61	0.41	0.18; 0.63	0.58	72	3
Water (g)	0.44	0.24; 0.60	0.48	0.34; 0.62	0.54	77	0
Food groups (g)							
Meat	0.44	0.22; 0.63	0.34	0.22; 0.55	0.96	74	5
Fish	0.14	0.07; 0.34	0.09	0.05; 0.22	0.49	50	15
Eggs	0.26	0.05; 0.50	0.26	0.06; 0.47	0.92	55	6
Dairy products	0.49	0.34; 0.68	0.78	0.48;1.08	0.69	87	3
Fruit	0.34	0.17; 0.53	0.50	0.20; 0.80	0.50	73	3
Vegetables	0.34	0.15; 0.48	0.27	0.11; 0.43	0.51	59	2
Nuts/seeds	0.49	0.26; 0.72	0.46	0.29; 0.64	0.93	75	6
Potatoes	0.45	0.24; 0.72	0.31	0.18; 0.45	0.74	67	3
Legumes	0.34	0.07; 0.67	0.19	0.08; 0.31	0.46	83	16
Grains	0.24	0.08; 0.40	0.20	0.03; 0.38	0.31	65	6
Bread	0.53	0.34; 0.69	0.66	0.44; 0.87	0.81	73	1
Alcoholic beverages	0.78	0.55; 0.90	0.63	0.52; 0.74	1.08	86	1
Non-alcoholic beverages	0.70	0.56; 0.82	0.57	0.45; 0.70	0.85	77	0
Sugar/sweets	0.42	0.24; 0.65	0.46	0.25; 0.68	0.86	65	1
Cakes/cookies	0.38	0.16; 0.58	0.27	0.13; 0.42	0.79	74	5
Fast food/snacks	0.34	0.10; 0.65	0.14	0.06; 0.23	1.08	57	7
Soups	0.28	0.09; 0.49	0.22	0.06; 0.34	1.14	75	6

* Based on log-transformed variables for macro- and micronutrients.

## Supplementary Materials

The following are available online at https://www.mdpi.com/2072-6643/12/7/1998/s1, Table S1: Mean intake (standard deviation) of energy, macronutrients and dietary fibre based on the FFQ of the 88 validation study participants (those with a completed food frequency questionnaire as well as three 24-hour dietary recalls) and all 1399 participants (those with complete food frequency questionnaire data) of the Longitudinal Aging Study Amsterdam.
