# Supplementary material for: Relative Validity of the HELIUS Food Frequency Questionnaire for Measuring Dietary Intake in Older Adult Participants of the Longitudinal Aging Study Amsterdam
Source: Nutrients. 2020 Jul 5;12(7):1998. doi: 10.3390/nu12071998 (PMC7400819; doi:10.3390/nu12071998)
Supplement: Supplementary file 1 [file nutrients-12-01998-s001.pdf]

Table S1. Mean intake (standard deviation) of energy, macronutrients and dietary fibre based on the FFQ of the 88 validation study participants (those with a completed food frequency questionnaire as well as three 24-hour dietary recalls) and all 1399 participants (those with complete food frequency questionnaire data) of the Longitudinal Aging Study Amsterdam.

**Characteristic**	**Validation sample**	**Total FFQ sample**
N	88	1399
Energy (kcal)	2097 (579)	2100 (582)
Total carbohydrates (g)	212.0 (72.7)	214.8 (66.7)
Mono/di-saccharides (g)	102.2 (47.3)	100.8 (40.5)
Total protein (g)	81.1 (24.2)	79.9 (23.7)
Total fat (g)	80.5 (28.9)	80.4 (28.4)
Dietary fibre (g)	22.2 (7.0)	22.9 (7.2)

FFQ=food frequency questionnaire
